# Impacted and Fractured Biliary Basket: A Second Basket Rescue Technique

**DOI:** 10.1155/2016/6210646

**Published:** 2016-05-11

**Authors:** Mohammed Amine Benatta, Ariane Desjeux, Marc Barthet, Jean Charles Grimaud, Mohamed Gasmi

**Affiliations:** ^1^Digestive Endoscopy Unit, Central Hospital of Army (HCA), 16000 Algiers, Algeria; ^2^Gastroenterology Department, North Hospital, University of the Mediterranean, 13000 Marseille, France

## Abstract

A 59-year-old woman was treated with ERCP, ES, and biliary plastic stent, for large and multiple common bile duct stones. During a second ERCP basket extraction was impacted with a round entrapped stone. The basket handle was cut off; a metal sheath of extraendoscopic lithotriptor was advanced over the basket. The mechanical lithotripsy was complicated with basket traction wires fracturing, without breakage of the stone. A rescue standard basket was pushed until it caught the basket/stone complex. Using this method disengagement of the whole fractured basket/stone complex was achieved without need of surgery. It is the third case reported in the English literature.

## 1. Introduction

Endoscopic retrograde cholangiopancreatography (ERCP) with endoscopic sphincterotomy (ES) and stone extraction using a Dormia basket or balloon catheters is the best choice for management of choledocholithiasis. Difficulties during stone removal occurs when the stone is hard and large (>1 cm) or when there is discrepancy between the stone size and diameter of the distal bile duct [1]. Several lithotripsy techniques as mechanical lithotripsy, electrohydraulic probe lithotripsy, extracorporeal shock wave lithotripsy, and laser lithotripsy may be used. Furthermore plastic or metallic stenting efficiency has been proved. Impaction of a biliary basket due to a hard stone is well known and reported in 0.8–6% of cases [2]. With the mechanical lithotripsy using Soehendra lithotriptor, stones captured within an impacted basket are crushed or the Dormia basket wires broken to release the trapped basket [3]. Rapid removal of impacted basket with entrapped stone in the distal common bile duct (CBD) with fractured basket traction wires is obligatory and its endoscopic management may be highly challenging. We report here a rescue second basket method leading to disengagement of the whole fractured basket/stone complex. It is the third case reported in the English literature, at our knowledge.

## 2. Case Report

**Figure 1:**
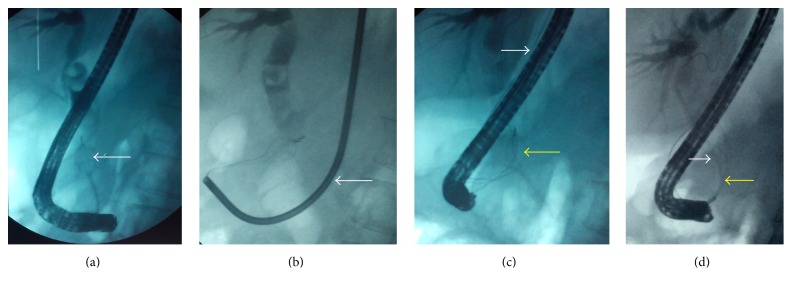
(a) The entrapped stone of almost 20 mm diameter and the impacted basket (arrow). (b) The metal sheath of extraendoscopic lithotriptor advanced to crush the stone (arrow). (c) The fracturing central traction wire (white arrow) and the basket without stone breakage (yellow arrow). (d) The basket/stone complex (white arrow) caught in the rescue standard basket (yellow arrow).

A 59-year-old woman was admitted in our unit for a second planned ERCP to complete biliary stones extraction. She was treated initially with ERCP, ES, and biliary plastic stent, for large and multiple common bile duct stones, 3 months earlier. Extending of the ES was performed prior to the stone extraction. Unfortunately the Dormia basket advanced within the common bile duct became impacted with a round entrapped stone of almost 20 mm in diameter Figure 1(a) and could not be disengaged. For endoscopic removal, the impacted Dormia basket's handle was cut off, the polyethylene sheath covering the basket removed making the central wire exposed and the duodenoscope withdrawn. To crush the stone, a metal sheath of extraendoscopic lithotriptor was advanced over the Dormia basket's central wire and attached to a Soehendra type mechanical lithotriptor handle, under a fluoroscopic control in Figure 1(b). But on cranking the lithotriptor, the central wire of the Dormia basket severed at the proximal end outside the oral cavity, without complete stone crushing Figure 1(c). Thereafter the lithotriptor was withdrawn and the duodenoscope reintroduced. A second Dormia basket was advanced until it caught the basket/stone complex Figure 1(d). Subsequently, by pulling downwards the whole second Dormia basket, the fractured Dormia basket and the stone, the fractured basket/stone complex was disengaged and the partially crushed stone removed. The patient recovered uneventfully and was discharged few hours later.

## 3. Discussion

If a biliary stone cannot be extracted by standard techniques, extraendoscopic mechanical lithotripsy usually solves the problem. A fractured Dormia basket with a captured stone is an unusual complication during extraction and its rapid removal is obligatory. In the past, surgical intervention was the standard method. Various nonsurgical techniques to release the impacted stone and basket have been reported. Spontaneous passage of the impacted basket and stone after successful biliary stent placement [4], extraendoscopic mechanical lithotripsy, extracorporeal shock wave lithotripsy [5], endoscopic pulse-dye laser [6, 7], and transhepatic choledochoscopic lithotripsy [8] have been reported. Extraendoscopic mechanical lithotripsy was the unique available method in our unit. A second Dormia basket which is widely available and less expensive was immediately and successfully used, in our case. In two similar cases of failure due to severed basket's central wire a rescue second basket was used to disengage the impacted basket from the stone. Only the tip of the impacted basket was caught with the second basket [1, 3] and in one of them surgery was required later to clear the stone [1]. At our knowledge the rescue second basket method we reported here is the third case in the English literature and the second one where disengagement of the whole fractured basket/stone complex was achieved without surgery. This simple method may be used as first line rescue technique before any invasive procedures.
